# Differential transcript abundance in adipose tissue of mature beef cows during feed restriction and realimentation

**DOI:** 10.1371/journal.pone.0194104

**Published:** 2018-03-13

**Authors:** Hannah Crocker Cunningham, Kristi M. Cammack, Kristin E. Hales, Harvey C. Freetly, Amanda K. Lindholm-Perry

**Affiliations:** 1 University of Wyoming, Laramie, WY, United States of America; 2 South Dakota State University West River Ag Center, Rapid City, SD, United States of America; 3 USDA, ARS, U.S. Meat Animal Research Center, Clay Center, NE, United States of America; INIA, SPAIN

## Abstract

Feed costs account for over 70% of the annual expenditures in cow/calf production. During the production year the cow uses nutrients to support conceptus growth, milk production, work (grazing and locomotion), and maintenance requirements. The majority of the nutrients are used to support maintenance. Substrate cycling has been identified as one of the major contributors toward energy expenditure associated with maintenance in mature cows. The objective of this study was to determine whether beef cows that differ in the efficiency of weight gain differ in the relative abundance of transcripts for metabolic regulation in adipose tissue. Mature beef cows were subjected to feed restriction followed by ad libitum feed. Adipose tissue from twelve cows with high (n = 6) and low (n = 6) gain based on growth performance during the ad libitum feeding period was evaluated for transcriptome expression differences. A total of 496 genes were differentially expressed and passed Bonferroni correction for the animals with greater gain between restriction and realimentation and 491 genes were differentially expressed among animals with lesser gains between feed restriction and realimentation. Of these two differentially expressed gene lists, 144 genes were common between animals with greater and those with lesser gain. Enriched biological processes included the TCA cycle, oxidative phosphorylation, respiratory electron transport chain and fatty acid metabolic processes. Specific to adipose tissue of low gaining animals was glycolysis and to high gain animals was coenzyme, steroid, cellular amino acid, nitrogen compound metabolic processes, and sensory perception. The oxidative phosphorylation, mitochondrial dysfunction and cholesterol biosynthesis pathways were commonly associated with the high gain animals between feed restriction and realimentation, as well as with the low gaining animals between the two time points. Unique to the high gaining animals were valine degradation and LPS/IL-1 mediated inhibition of RXR function pathways. In this discovery study, genes involved in lipid metabolism, mitochondrial respiration and oxidative phosphorylation pathways appear to be critical to mature cows during times of abundant feed after feed restriction.

## Introduction

With feed costs accounting for a large portion of total cow operating costs, increased feed prices, and decreased land availability due to alternative land use, supplying ample nutrition to livestock has become increasingly difficult. The potential effects that periods of nutrient restriction may have on animal performance, both in the short and long term, are critical to understand in order for producers to optimize production. Unfortunately, the metabolic changes associated with periods of nutrient restriction and realimentation, as well as the downstream effects on production, remains largely unknown in beef cattle.

Beef cows often experience periods of nutrient restriction, especially during times of the production cycle when requirements are high (i.e. gestation and lactation) and the quality and quantity of feedstuffs is low. During periods of feed restriction, the animal responds phenotypically by changes that may be driven by metabolic changes on the tissue and organ level. Yang et al. [1] reported in long-term feed-restriction of lambs, adipose tissue responded by inhibiting lipogenesis and stimulating lipolysis in an effort to avoid starvation. These specific changes include a decrease in lipogenic gene expression across several fat depots and a decrease in regulatory genes of lipogenesis as well [1]. Periods of feed restriction are often followed by an increase in plane of nutrition due to temporal changes in nutrient availability. This increase in available nutrients following feed restriction results in a period of compensatory growth. This compensatory growth results in an increase in efficiency of growth; however, this is temporal and decreases exponentially over time [2,3]. Freetly et al. [4] have described acute adaptation of heat production associated both with feed restriction and realimentation occurring within 7 days of the transition, suggesting responses in metabolic rate. Feed restriction was associated with an initial rapid decrease in heat production and realimentation was associated with a rapid increase in heat production both followed by long term adaptation [4]. These evident shifts in metabolic rate, suggest cellular and tissue responses to energy availability, yet the mechanisms driving these responses remain largely unknown.

In beef cows, nutrients are required to support growth and development of conceptus, milk production, work, and maintenance. Caton and Dhuyvetter [5] reported that maintenance can range from 60–90% of total herd energy expenditures. Cows are considered to meet maintenance requirements when they have a net energy balance of zero and retain no energy. Periods of nutrient restriction and realimentation result in a net negative or positive energy balance, respectively. Cattle respond differently to periods of nutrient restriction and recovery. The metabolic processes perturbed by nutrient restriction may highlight areas where some cattle more efficiently respond to periods of nutritional stress. Thus, we hypothesize that metabolic differences may exist in subcutaneous adipose in response to a period of nutrient restriction followed by realimentation, between cattle divergent for body weight gain. The objective of this research program is to determine if beef cows that differ in efficiency of weight gain during ad libitum feeding differed in the relative abundance of transcripts for metabolic regulation in adipose tissue.

## Methods

### Animals

The U.S. Meat Animal Research Center (USMARC) Animal Care and Use Committee reviewed and approved all animal procedures. The procedures for handling cattle complied with the Guide for the Care and Use of Agricultural Animals in Agricultural Research and Teaching [6]. Crossbred cows (n = 121) that were the result of sampling sires used in the industry were used in the study. Angus, Hereford, and MARC III composite (¼ Angus, ¼ Hereford, ¼ Pinzgauer, ¼ Red Poll) cows were bred by artificial insemination to Angus, Hereford, Simmental, Limousin, Charolais, Gelbvieh and Red Angus bulls. The F_1_ bulls from these matings that had Angus and Hereford dams were mated to F_1_ cows from these matings in multiple-sire pastures to produce 2-, 3- and 4-breed cross progeny. The resulting female progeny were kept and raised to have their first calf as 2-year olds. At 5 years of age, cows were not bred, and were moved to an individual feed intake facility equipped with Calan Gates (American Calan, Northwood NH) the week after their calves were weaned. Non-pregnant, lactating, 5-year old cows were used to not confound the physiology associated with pregnancy, lactation, or growth. The initial feeding period was used to allow cows to come to an energetic maintenance. Weight gain is a normal part of the production cycle and there is variation in the rate that mature cows gain weight. Extremes in weight gain were chosen to study variation in adipose tissue among cattle that differed in rates of gain offered the same resources. Cows were fed a ration that contained as a percent of DM 27.0 ground alfalfa hay, 5.0 corn, 67.8 corn silage, and 0.2 salt. Twenty-one days after weaning, cows were weighed on two consecutive days. Body weight was averaged and feed offered to provide 120 kcal ME/kg BW^0.75^. Cows were fed the same amount of feed for 112 days. At 112 days, cows had ad libitum access to the same ration for an additional 98 days. Cows were fed once a day and feed refusal were measured weekly. During the feed restriction, cows were weighed on days 0, 1, 14, 28, 56, 84, 111, and 112. During the ad libitum period, cows were weighed on days 0, 14, 28, 42, 56, 70, 84, 97, and 98. Individual BW was regressed on time during the ad libitum period using a quadratic equation, and BW gain over the study was calculated from the regression equation.

### Tissue samples

Adipose biopsies were taken 105 days after the start of the feed restriction, and 49 days after the start of the ad libitum feeding period. Adipose tissue collections occurred over 3 days. Approximately 40 animals were sampled each day from 07:00 to 13:00 hours. Animals were allowed access to feed and water up until they were moved in small groups of 8–9 animals to the barn for sample collection and were not kept in the holding area prior to sampling for longer than 1 hour. The sample site was scrubbed with betadine and rinsed with water. Twenty milliliters of lidocaine was injected subcutaneously at the incision site, and a 5-cm incision was made through the skin with a scalpel above the fat pad between the hooks and pins. Adipose tissue sample was harvested with iris scissors. The sample was immediately frozen in liquid nitrogen and stored at -80° C. The wound was closed with Braunamid 4 USP (B. Braun AESCULAP, Germany) suture, and sutures were removed 14 days later. The sample taken during restriction was from the left side of the cow, and the sample take during ad libitum feeding was from the right side of the animal.

Six cows with the greatest and 6 cows with the least BW gain during the ad libitum period were selected and adipose tissue from those 12 animals was processed for microarray analysis. These 12 cows were selected to represent the largest difference in gain during the ad libitum period. Breed diversity among the high gain animals was: 25% Hereford, 16.7% Angus, 16.7% MARCIII, 12.5% Simmental, 12.5% Gelbvieh, and 16.7% Red Angus. Breed composition among the low gain animals was: 25% Hereford, 16.7% Angus, 16.7% MARCIII, 4.2% Simmental, 4.2% Limousin, 4.2% Charolais, 4.2% Gelbvieh, and 25% Red Angus.

### RNA extraction

Total RNA was extracted from 50–100 mg of tissue by homogenization with TriPure reagent (Roche, Indianapolis, IN) following the manufacturer’s protocol with the exception that the time of centrifugation following the addition of chloroform was 20 minutes, rather than 15 minutes. The RNA pellets were resuspended in sterile water and RNA was stored at -80°C until further processing. Purified RNA was quantified using a NanoDrop 8000 spectrophotometer (Thermo Scientific, Wilmington, DE, USA). Absorbance was measured by spectrophotometry at 260 and 280 nm. Average 260/280 measurements were 1.94 and 1.96 for adipose samples collected from cows at feed restriction and realimentation, respectively. The quality of total RNA was determined by running samples on a RNA 6000 LabChip kit (Agilent Technologies, Santa Clara, CA, USA) with the Agilent 2100 Bioanalyzer. Average RIN values were 6.6 and 7.2 for adipose tissue samples collected from cows at feed restriction and realimentation.

### Microarray

To assess the differential expression of genes in adipose tissue, the Affymetrix GeneAtlas System (Santa Clara, CA, USA) in conjunction with Bovine 1.1ST array strips were used. Briefly, RNA purity and yield was confirmed by spectrophotometry. 100ng of total RNA and controls were converted to single stranded cDNA, then fragmented and TdT labeled according to the WT Expression Kit manufacturer’s instructions (Ambion®, Life Technologies Corporation). Chips were washed and stained in the GeneAtlas Fluidics station. Chips were immediately imaged in a calibrated Affymetrix GeneAtlas scanner. CEL formatted files were converted to CHP files with Affymetrix Expression Console software [7]. Guanine Cytosine Count Normalization (GCCN) and Signal Space Transformation (SST) algorithms were applied to the microarray data. The GCCN program normalizes the signal of the probes by GC content. The SST algorithm was used to stretch the signal intensity distribution in order to decompress the fold change ratios. Samples were then subjected to Robust Multichip Analysis (RMA) for further normalization and analysis of the microarrays [8]. Transformed data was analyzed with the Affymetrix Expression Console and Transcription Analysis Console software. Genes were considered differentially expressed when a *P*-value of <0.05 and a fold change of >2 was obtained. A correction for multiple testing (Bonferroni correction) was also applied by multiplying the *P*-value by the total number of genes tested (n = 24,341). Data files were deposited in the NCBI gene expression omnibus (GEO) and can be accessed by Series record GSE94746.

### Panther gene ontology enrichment analysis

The biological functions of differentially expressed genes were determined using the PANTHER classification system (Version 11.0). Enrichment analysis of gene function was performed using PANTHER’s implementation of the binomial test of overrepresentation. Significance of gene ontology (GO) terms was assessed using the default Ensembl *Bos taurus* GO annotation as background for the enrichment analysis. Data from PANTHER was considered statistically significant at a Bonferroni corrected *P*-value < 0.05.

### Ingenuity pathway analysis

The Ingenuity ® Pathway Analysis (IPA®, QIAGEN Redwood City, www.qiagen.com/ingenuity) tool depicts gene interactions on a system level. Results of this software were based on human-interpretation of peer-reviewed gene and protein interaction literature. The Ingenuity Knowledge Base contains this database of known interactions and used the imported data Gene symbol to assess differentially expressed genes based on the log base 2 fold change ratios. The Top Canonical Pathways generated by IPA were utilized to analyze the 6 comparisons.

## Results

### Phenotypic data

**Fig 1:**
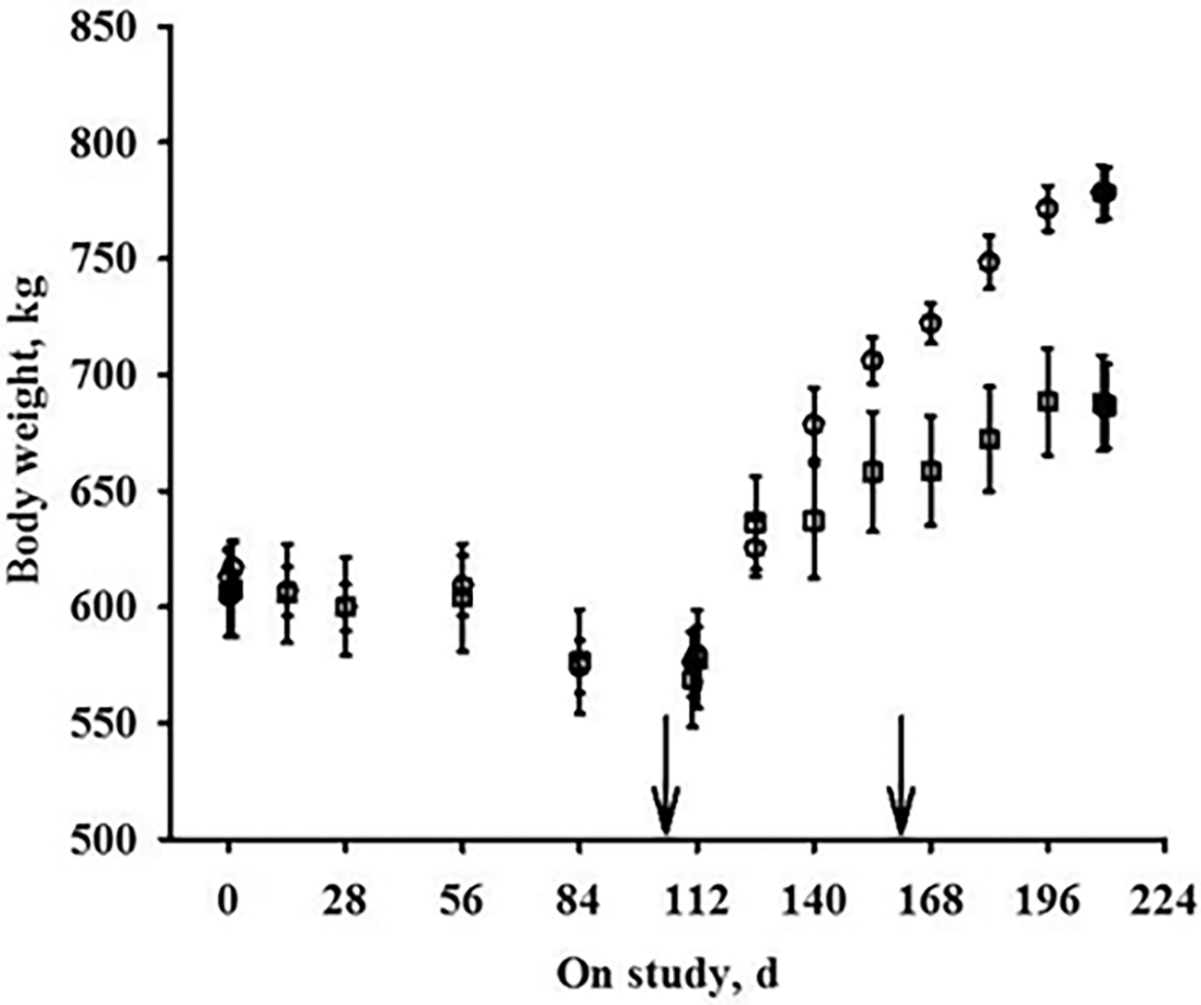
Body weight gain of cows with greater gain or lesser gain.

The phenotypic data for cows with high and low gain is presented in Fig 1. Cows selected for the high gain group had average gains of 2.01kg ± 0.08 and average daily dry matter intake of 17.2 ± 1.5kg (*P*<0.0001). Cows in the low gain group had an average gain of 0.99kg ± 0.14 and average daily dry matter intake of 13.3 ± 1.1kg (*P* = 0.0004). The high gain animals indicate those that respond most favorably in terms of compensatory gain; however, it should be noted that the cows selected for greater gain during realimentation also had greater feed intake during this period.

### Gene expression results

#### Feed restriction study (high versus low gain comparison)

A total of 113 genes were differentially expressed in the adipose tissue from the feed restriction study between the high and low gain cows (S1 Table). Of those, 45 genes were up-regulated and 68 were down-regulated. Thirteen of these genes were not annotated in the genome assembly. None of these differentially expressed genes passed Bonferroni correction for multiple testing. Using the list of DEGs, Panther generated the biological process of lipid metabolism as over-represented with a fold enrichment of 5.1.

#### Ad libitum study (high versus low gain comparison)

Sixty genes were differentially expressed between the high and low gain from the ad libitum feeding study. Of these, 33 genes were up-regulated and 27 down-regulated genes; six of these genes were not annotated (S2 Table). Following the Bonferroni correction, none of the genes remained differentially expressed.

#### High gain animals (feed restriction to ad libitum comparison)

A total of 2,878 genes were differentially expressed in the adipose tissue of the high gain cows between feed restriction and ad libitum studies (S3 Table). Of those genes, 2,295 were up-regulated and the remaining 583 genes were down-regulated. After correcting for the number of gene probes on the array (24,341), a total of 496 genes remained significantly different across these two groups.

GO terms identified by Panther as over-represented among the DEG list included TCA cycle oxidative phosphorylation, respiratory ETC, and coenzyme, fatty acid, steroid, cellular amino acid and nitrogen compound metabolic processes (Table 1). Those under-represented were sensory perception genes. IPA identified the following pathways: oxidative phosphorylation, mitochondrial dysfunction, valine degradation I, LPS/IL-1 mediated inhibition of RXR function, and superpathway of cholesterol biosynthesis (Table 2). A list of genes identified with biological functions in mitochondrial respiration pathways are shown in Table 3.

**Table 1:** Biological processes over-represented among the list of DEG (*P* < 0.05) identified in adipose tissue after feed restriction and realimentation for cows with greater body weight gain.

Term^1^	# Identified^2^	# Expected^3^	Fold Enrichment^4^	Bonferroni Corrected *P*-value
Tricarboxylic acid cycle	12	2.6	4.6	4.43E-03
Oxidative Phosphylation	26	6.5	4	1.30E-06
Respiratory ETC	40	16.8	2.4	2.18E-04
Coenzyme metabolic process	26	11	2.4	1.99E-02
Fatty Acid Metabolic Process	39	19	2	8.01E-03
Steroid Metabolic Process	37	18.5	2	2.29E-02
Cellular Amino Acid Metabolic Process	50	26.6	1.88	6.76E-03
Nitrogen Compound Metabolic Process	255	205	1.25	4.42E-02
Sensory Perception of Smell^5^	30	69.5	0.43	1.27E-05

^1^ Biological process enriched for in the list of DEG genes.

^2^ Number of DEG identified within the biological process.

^3^ The number of genes that expected based on the reference genes.

^4^ Fold Enrichment of the genes observed in the list of DEG genes that is over the expected.

^5^ The biological process of sensory perception of smell was represented by fewer genes than expected.

**Table 2:** Ingenuity pathway analysis of differentially expressed genes (*P* < 0.05) from adipose tissues from cows with greater gain between feed restriction and realimentation sample collection, and for cows with lesser gain between feed restriction and realimentation.

Comparison	Top Canonical Pathway	*P*-value	#DEG Represented
High Gain	Oxidative Phosphorylation	3.27E-25	51
	Mitochondrial Dysfunction	4.09E-21	63
	Valine Degradation I	1.01E-05	10
	LPS/IL-1 Mediated Inhibition of RXR Function	1.48E-05	45
	Superpathway of Cholesterol Biosynthesis	2.08E-05	12
Low Gain	Oxidative Phosphorylation	1.04E-23	45
	Mitochondrial Dysfunction	7.44E-22	57
	Superpathway of Cholesterol Biosynthesis	1.70E-07	13
	Cholesterol Biosynthesis I	3.55E-06	8
	Cholesterol Biosynthesis II (via 24,25-dihydrolanosterol)	3.55E-06	8

**Table 3:** Differentially expressed genes with mitochondrial energy production functions identified in cows with higher gain between feed restriction and realimentation.

Gene^1^	Gene Name	ETC Function^2^	Fold Change^3^	*P*-value ^4^ (Corrected)
*ATP5A1*	ATP synthase alpha subunit 1, cardiac muscle	Complex V	1.08	0.02
*ATP5C1*	ATP synthase gamma polypeptide 1	Complex V	1.11	0.05
*ATP5F1*	ATP synthase subunit B1	Complex V	1.09	0.009
*ATP5G3*	ATP synthase subunit C3 (subunit 9)	Complex V	1.17	0.02
*ATP5H*	ATP synthase subunit d	Complex V	1.08	0.005
*ATP5O*	ATP synthase O subunit	Complex V	1.11	0.02
*ATPIF1*	ATPase inhibitory factor 1	Complex V	1.10	0.001
*COX7A2*	cytochrome c oxidase subunit VIIa polypeptide 2 (liver)	Complex IV	1.12	0.007
*NDUFA10*	NADH dehydrogenase 1 alpha subcomplex, 10	Complex I	1.20	0.008
*NDUFA13*	NADH dehydrogenase 1 alpha subcomplex, 13	Complex I	1.18	0.0003
*NDUFA2*	NADH dehydrogenase 1 alpha subcomplex, 2	Complex I	1.13	0.02
*NDUFA4*	NADH dehydrogenase 1 alpha subcomplex, 4	Complex I	1.10	0.01
*NDUFB5*	NADH dehydrogenase 1 beta subcomplex, 5	Complex I	1.15	0.0003
*NDUFS2*	NADH dehydrogenase Fe-S protein 2	Complex I	1.11	0.02
*NDUFS5*	NADH dehydrogenase Fe-S protein 5	Complex I	1.09	0.005
*NDUFS6*	NADH dehydrogenase Fe-S protein 6	Complex I	1.13	0.05
*SDHD*	succinate dehydrogenase complex, subunit D, integral membrane protein	Complex II	1.12	0.0003
*UQCR11*	ubiquinol-cytochrome c reductase, complex III subunit XI	Complex III	1.19	0.0005
*UQCRH*	ubiquinol-cytochrome c reductase hinge protein	Complex III	1.11	0.02
***FH***	**fumarate hydratase**	TCA	1.12	0.010

^1^ All genes in Complex I-V were also identified as differentially expressed in low gain animals, but they were not significant after correction for multiple testing, except for fumerate hydratase (FH) shown in bold, which was not identified as differentially expressed in low gain animals at P < 0.05.

^2^ Components of the TCA and oxidative phosphorylation pathways that each of the DEG is associated with.

^3^ Fold change is the log2 fold change difference between ad libitum and feed restriction studies. These genes were more highly transcribed during the ad libitum study.

^4^ The P shown was corrected by multiplying the nominal P by the number of genes tested (24,431).

#### Low gain animals (feed restriction to ad libitum comparison)

There were 2,474 genes differentially expression in the low gain adipose samples compared between feed restriction and ad libitum studies (S4 Table). Of those, 2,041 genes were up-regulated and 433 were down-regulated. Following the Bonferroni correction, 491 genes remained differentially expressed with 475 up-regulated and 16 down-regulated. A total of 144 genes were common between both high gain and low gain animals (S5 Table). The gene ontology terms identified as over-represented were TCA cycle, oxidative phosphorylation, glycolysis, respiratory ETC, and fatty acid metabolism (Table 4). The DEG with biological functions within lipid metabolism pathways for both high and low gain animals are shown in Table 5. IPA identified the following pathways over-expressed from the list of DEG: oxidative phosphorylation, mitochondrial dysfunction, superpathway of cholesterol biosynthesis, cholesterol biosynthesis I and cholesterol biosynthesis II (Table 2).

**Table 4:** Biological processes of over-represented among the list of DEG (P < 0.05) identified in adipose tissue after feed restriction and realimentation for cows with lower body weight gain.

Term^1^	# Identified^2^	# Expected^3^	Fold Enrichment^4^	Nominal *P*-value
Tricarboxylic Acid Cycle	11	2	5.5	2.13E-03
Oxidative Phosphorylation	25	5	5	3.50E-08
Glycolysis	12	3	4	1.25E-02
Respiratory Electron Transport Chain	35	13	3	6.85E-05
Fatty Acid Metabolic Process	37	15	2.5	1.54E-04

^1^ Biological process enriched for in the list of DEG genes.

^2^ Number of DEG identified within the biological process.

^3^ The number of genes that expected based on the reference genes.

^4^ Fold Enrichment of the genes observed in the list of DEG genes that is over the expected.

**Table 5:** Genes with biological functions in lipid metabolism pathways identified as differentially expressed for high and low gain animals.

Gene^1^	Gene Name	Fold Change Low Gain^2^	*P*-value	Fold Change High Gain^2^	*P*-value
*ACACA*	acetyl-CoA carboxylase alpha	4.58	0.000078	9.32	**0.000004**
*ACADS*	acyl-CoA dehydrogenase, C-2 to C-3 short chain	1.21	0.0019	2.60	0.000084
*ACADSB*	acyl-CoA dehydrogenase, short/branched chain	1.04	0.0021	1.93	0.00039
*ACAT1*	acetyl-CoA acetyltransferase 1	1.05	0.0043	1.01	0.00069
*ACAT2*	acetyl-CoA acetyltransferase 2	2.11	0.00071	1.37	0.0019
*ACLY*	ATP citrate lyase	41.07	0.000035	**29.65**	**0.000044**
*ACO2*	aconitase 2, mitochondrial	1.47	0.012	2.17	0.00035
*ACOX1*	acyl-CoA oxidase 1, palmitoyl			1.01	0.024
*ACOX2*	acyl-CoA oxidase 2, branched chain	2.19	0.00031	2.27	0.000065
*ACSL1*	acyl-CoA synthetase long-chain family member 1			**1.73**	**0.00002**
*ACSL4*	acyl-CoA synthetase long-chain family member 4	1.10	0.0019	1.83	0.014
*ACSL5*	acyl-CoA synthetase long-chain family member 5; long-chain-fatty-acid—CoA ligase 5-like	2.16	0.0033	1.27	0.020
*ACSS2*	acyl-CoA synthetase short-chain family member 2	4.20	0.00012	**10.27**	**0.000026**
*ACSS3*	acyl-CoA synthetase short-chain family member 3			1.06	0.071
*AGPAT2*	1-acylglycerol-3-phosphate O-acyltransferase 2 (lysophosphatidic acid acyltransferase, beta)	2.00	0.0038	7.52	0.000087
*ALDH2*	aldehyde dehydrogenase 2 family (mitochondrial)	1.20	0.0014	1.29	0.0013
*ALDOA*	aldolase A, fructose-bisphosphate	**1.55**	**0.000031**	**1.68**	**0.000047**
*ANGPTL4*	angiopoietin-like 4	0.10	0.00035	0.07	0.00018
*APOE*	apolipoprotein E	2.23	0.0096	1.75	0.011
*CPT1A*	carnitine palmitoyltransferase 1A (liver)	**0.14**	**0.000028**	0.07	0.00011
*CPT1B*	carnitine palmitoyltransferase 1B (muscle)	0.22	0.00086	0.21	0.00086
*CPT2*	carnitine palmitoyltransferase 2			1.67	0.0016
*ECHS1*	enoyl CoA hydratase, short chain, 1, mitochondrial	2.31	0.00086	**3.58**	**0.000031**
*ECI2*	enoyl-CoA delta isomerase 2	1.66	0.028	1.13	0.016
*EHHADH*	enoyl-CoA, hydratase/3-hydroxyacyl CoA dehydrogenase	1.04	0.0054	1.58	0.0010
*ELOVL4*	ELOVL fatty acid elongase 4	**0.17**	**0.000002**	**0.17**	**0.000036**
*ELOVL5*	ELOVL fatty acid elongase 5	3.29	0.00016	4.29	0.00029
*ELOVL6*	ELOVL fatty acid elongase 6	5.24	0.000085	**6.87**	**0.000018**
*FABP7*	fatty acid binding protein 7, brain			1.18	0.0084
*FASN*	fatty acid synthase	4.56	0.0016	**6.92**	**0.000029**
*G6PD*	glucose-6-phosphate dehydrogenase	2.16	0.00021	**3.68**	**0.000033**
*GPI*	glucose-6-phosphate isomerase			**5.54**	**0.000041**
*LPL*	lipoprotein lipase	1.02	0.00053	1.26	0.00027
*LSS*	lanosterol synthase (2,3-oxidosqualene-lanosterol cyclase)	3.14	0.00049	2.69	0.0012
*PCK1*	phosphoenolpyruvate carboxykinase 1 (soluble)	1.53	0.045	1.36	0.044
*PCK2*	phosphoenolpyruvate carboxykinase 2 (mitochondrial)	1.47	0.0031	5.24	0.000078
*PFKFB1*	6-phosphofructo-2-kinase/fructose-2,6-biphosphatase 1	1.37	0.013	1.21	0.0018

^1^ Human genome organization gene nomenclature committee gene symbol.

^2^ Fold change is the log2 fold change difference between ad libitum and feed restriction studies. Genes with values > 1 were up-regulated during realimentation, genes with values < 1 were down-regulated. Fold changes and P-values in bold represent values that were significant after Bonferroni correction.

## Discussion

Few studies to elucidate the molecular mechanisms that underlie feed restriction and realimentation in cattle have been published to date [2, 9]. The purpose of this discovery study was to examine the adipose transcriptome of mature cows with variation in body weight gain under conditions of feed restriction and realimentation. We hypothesized that we would identify gene expression differences among genes involved with lipid metabolism pathways. While we were able to identify pathways related to lipolysis and lipogenesis, other pathways involved in mitochondrial energy production were also identified among higher and lower gain animals when comparing them across nutritional treatment. It is important to note that there were differences in dry matter intake between the groups of selected animals (i.e., the animals with higher gain also had greater feed intake); thus, changes in gene expression may be driven by a combination of both phenotypes rather than by gain alone.

Due to the small collection size of adipose tissue collected by biopsy, we were unable to validate the expression of the DEG identified in this study using qRT-PCR. However, on different populations of animals, we have performed prior microarray assays on larger tissue samples, which allowed us to validate the DEG using real-time gene expression [10–13]. Under these circumstances, we were able to detect greater than 68% concordance between microarray and qRT-PCR gene expression.

It is of interest that the gene expression differences detected by analyses using phenotype (i.e., high versus low body weight gain) compared at either restricted state or ad libitum feeding did not pass FDR correction for multiple testing yet, differentially expressed genes detected by treatment (restriction vs. ad libitum) within group (high or low gain animals) retained DEG after FDR correction. Weight gain is a complex trait that is controlled by many genes that may have only small effects on the phenotype, as over 700 quantitative trait loci have been identified for ADG (https://www.animalgenome.org/cgi-bin/QTLdb/BT/index). There appear to be fewer genes or possibly genes with larger effects identified among animals undergoing feed restriction to realimentation. Interestingly, these genes tend to correspond to biological functions such as mitochondrial energy production pathways, fatty acid metabolism and propanoate metabolism pathways, and also genes involved in the ECM. Moreover, the physiological status of cows between maintenance (zero energy balance) and cows gain weight (positive energy balance) is much greater than two groups in positive energy balance.The adipose tissue compared between feed restriction and realimentation for either the high gain or the low gain animals revealed the gene ontology annotation enrichment for energy production via TCA cycle, oxidative phosphorylation, and respiratory ETC. The expression values for mitochondrial energy production genes were lower during feed restriction than during realimentation. This is consistent with data evaluating heat production as a proxy for metabolic rate in cows during restriction [4]. Heat production decreased in cows undergoing feed restriction in two phases, acute and chronic, with the acute phase occurring through day 7. The nutrient restriction in this study was designed to feed cows at slightly below maintenance for 112 days at which time they should be at a zero energy balance. We sampled adipose tissue as they were nearing this stage at 105 days. Assuming that mitochondrial energy production is, in part, responsible for a cow’s metabolic rate, the decrease in transcription of mitochondrial energy production genes during feed restriction emulates previous heat production data.

A recent study in bulls to examine the molecular mechanisms of compensatory gain in muscle tissue after a feed restriction event also produced over-representation of DEG involved in mitochondrial energy production [2]; however, the direction of the gene expression was opposite of the expression in this study. Specifically, we identified several NADH dehydrogenase, Q cytochrome c reductase, cytochrome c oxidase, and ATP synthase genes with higher expression among the animals after ad libitum feeding; while Keogh et al. [2] found these genes to be down-regulated. The gender and ages of animals in these two studies, the tissue type sampled, and the timing of sample collection was different between these two studies and may contribute to these differences. The biopsy during realimentation in this study was performed on bulls at 479 days old at 49 days after refeeding versus 15 days in the study by Keogh et al. [2]. We sampled mature 5-year-old cows at 49 days of refeeding to avoid any potential lag in molecular mechanism that might occur early during refeeding in adipose tissue. In lambs, visceral organs including blood, internal organs and attached fat gain mass more quickly after refeeding than body fat and non-visceral fat [14]. Tissues like muscle and adipose appear to require additional time for gain as indicated by a slower weight gain than visceral weight through 14 days of refeeding. Freetly et al. [4] also showed that there are acute and chronic phases of adaptation resulting in increases in heat production during realimentation. The transition to the chronic phase of heat production occurred by day 14, but was dependent upon realimentation diet level.

The up-regulation of energy related pathways in mature cows may be related to an increase in lipid substrates derived from an increase in intake by these animals during realimentation. In support of this was the increase in expression of several genes associated with lipogenesis (i.e., ACACA, THRSP, GPAM, and LPL) and fatty acid metabolic process (FASN, ACOX2, LIPE, DECR1, ACSL4, ACSL5) after realimentation in both greater and lower gain groups of animals. A study by Crookenden et al. [15] showed that the genes ACACA, THRSP, GPAM, and LPL were up-regulated in the adipose tissue of dairy cows with higher metabolizable energy intake. The expression of the genes ACACA, THRSP, and GPAM has been associated with adipose deposition [16,17]. A feed restriction study in sheep showed a decrease in the expression of ACACA and FASN compared to control animals [1]. These two genes act in the de novo fatty acid synthesis pathway. We do not conclusively show that these genes are in lower abundance with feed restriction in cattle, as we did not measure control animals, but compared to realimentation, their relative expression is lower, supporting elevated lipogenesis, as opposed to lipolysis, is occurring in cows during realimentation. The feed intake of the mature cows during realimentation appears to exceed the intake requirements for maintenance and protein synthesis.

We identified DEG involved in fatty acid synthesis (i.e., ACACA, ACLY, FASN, ELOVL4, SLC27A3), peroxisome β-oxidation (i.e., ACOX1, ACOX2, EHHADH), gluconeogenesis (i.e., PCK1, PCK2), and ketogenesis (ACAT1). These genes were all up-regulated in both higher and lower gaining cows upon refeeding. Genes involved in mitochondrial β-oxidation of fatty acids (i.e., ACSL1, EHHADH) were also differentially expressed, and transcribed in higher abundance upon refeeding. Also of interest was the expression of LPL and its inhibitor ANGPTL4. The expression of LPL was lower, and expression of ANGPTL4 was higher in the cows during the feed restriction study suggesting that triglyceride hydrolysis in adipose tissue was reduced in cows on restricted feed. Ultimately, genes expressed during the nutrient restricted period should result in overall decrease in lipogenesis.

Several of the over-represented genes were involved in propanoate metabolism and were down-regulated in the nutrient restricted tissues compared to the tissue collected during ad libitum feeding. Propionate is one of the primary VFA produced by rumen microbes and is the main precursor for glucose synthesis via gluconeogenesis in the hepatic tissue of ruminants. In ruminants VFA are produced and absorbed in the gastrointestinal tract, the rumen primarily [18]. In adipose tissue it has been suggested that propionate may regulate adipogenesis and adipokine release via the two primary G-protein coupled receptors [19]. Our data showed that aldehyde dehydrogenase 6 family, member A1 (*ALDH6A1*), a key enzyme in propionate metabolism, was down-regulated during feed restriction across high gain and low gain animals and this gene is critical for many steps throughout the propionate pathway. The majority of the other genes in this pathway were also down-regulated suggesting that the nutrient restriction resulted in a shift in gene expression that overall reduced the metabolism of propionate, potentially via a lack of available propionate in a nutrient restricted state. This pathway is critical for energy metabolism and a shift in genes critical to this pathway suggests that tissue-wide nutrient restriction induced phenotype is induced by altered expression in genes throughout this metabolic pathway.

Genes involved in cholesterol biosynthesis were identified in this study as differentially expressed among both higher and lower gaining animals between feed restriction and realimentation. The genes HMGCS1, HMGCR, LSS and CYP51A1 were all transcribed in higher abundance during realimentation. These same genes were also found differentially expressed in the liver of chickens that were refed after a short-term feed restriction [20]. Cholesterol is a component of the cell membrane and has been shown to be down-regulated in other species and tissues in response to fasting or feed restriction [20,21]. It may be a cellular response that can be spared to preserve energy when feed availability is scarce.

Three collagen genes (*COL1A1*, *COL1A2*, and *COL3A1*) involved in extracellular matrix remodeling were detected as up-regulated after realimentation among both high and low gain cows. COL1A1 has been identified as differentially expressed in various tissues like liver and rumen in several studies of feed efficiency in cattle [22–24]. Connective tissue and extracellular matrix proteins are found throughout adipose tissue. In cell culture it appears that adipose tissue uses energy to maintain the extra cellular matrix (ECM) [25]. The restructuring of the ECM is costly in terms of energetics [26] and the data in this study suggests that ECM is an important component of adipocyte alteration during realimentation.

There were some differences in the pathways identified for adipose tissue for animals with high gain and animals with lower gains as they transitioned to the ad libitum diet. The high gain animals produced genes DE in the valine degradation and LPS/IL-1 mediated inhibition of RXR function pathways. Valine degradation, and also leucine and isoleucine degradation pathways, prevents the accumulation of toxic branched chain amino acids [27]. The valine degradation pathway with some of the same genes DEG (*ACAT1*, *EHHADH*, *HMGCS1*) was also previously identified in chickens by Désert et al. [20]. The high gaining cows were consuming more feed than the low gain animals (*P* = 0.0004), perhaps driving up the regulation of this pathway due to an increase in substrate. Additionally, any changes in the rate of synthesis or degradation of amino acids can alter rate of gain [28]. Branched chain amino acids can also contribute to lipogenic acetyl-coenzyme A (AcCoA) levels in differentiated adipocytes [27]. The LPS/IL-1 mediated inhibition of RXR function pathway was detected due to the differential expression of genes like *CD14*, *SOD1*, *UGT2A3*, and several GST and CYP metabolism genes in the high gain animals.

This is the first study that we know of to examine the transcriptome changes in subcutaneous adipose tissue of mature cows under feed restriction and realimentation. Analyses comparing high and low gain phenotypes during each nutritional study produced no differentially expressed genes that withstood Bonferroni correction; however, analyses comparing high gain animals between treatments (and low gain animals between treatment) produced >490 genes that passed Bonferroni correction with *P* < 0.05. In addition, approximately 70% of the genes that were detected differed by group of animals, while many of the biological functions and pathways were the same. This suggests that on the system level adipose tissue of the mature cow responds similarly to nutrient restriction and realimentation, yet the specific transcripts responsible for these biological changes may differ between animals with higher compensatory gain response versus those with a lower response. The pathways critical to readjustment to an abundance of feed included mitochondrial energy production pathways, fatty acid metabolism and propanoate metabolism pathways, and also genes involved in the ECM. Further investigation, including validation of genes are necessary to confirm these discovery data in other populations of cows; however, these data provide a platform for continued work to identify key genes and associated biological function in adipose tissue associated with divergence in weight gain.

## Supporting information

S1 Table Differentially expressed genes between animals with lesser (n = 6) and greater (n = 6) gain during feed restriction. (XLSX)

S2 Table Differentially expressed genes between animals with lesser (n = 6) and greater (n = 6) gain during realimentation. (XLSX)

S3 Table Differentially expressed genes identified among animals with greater body wieght gain between feed restriction (n = 6) and realimentation (n = 6). (XLSX)

S4 Table Differentially expressed genes identified among animals with lesser body weight gain between feed restriction (n = 6) and realimentation (n = 6). (XLSX)

S5 Table Genes identified as differentially expressed between feed restriction and realimentation for cows with lesser and greater gain. (XLSX)
